# Fabrication of Microwave Devices Based on Magnetic Nanowires Using a Laser-Assisted Process

**DOI:** 10.3390/mi10070475

**Published:** 2019-07-16

**Authors:** Vivien Van Kerckhoven, Luc Piraux, Isabelle Huynen

**Affiliations:** 1 IMCN Institute, Université catholique de Louvain, 1 place Croix du Sud, 1348 Louvain-la-Neuve, Belgium; 2 ICTEAM Institute, Université catholique de Louvain, 3 place du Levant, 1348 Louvain-la-Neuve, Belgium

**Keywords:** microwave, ferromagnetic, laser processing, substrate integrated waveguide, nanowire

## Abstract

This paper compares two laser-assisted processes developed by the authors for the fabrication of microwave devices based on nanowire arrays loaded inside porous alumina templates. Pros and cons of each process are discussed in terms of accuracy, reproducibility and ease of fabrication. A comparison with lithography technique is also provided. The efficiency of the laser-assisted process is demonstrated through the realization of substrate integrated waveguide (SIW) based devices. A Nanowired SIW line is firstly presented. It operates between 8.5 and 17 GHz, corresponding to the first and second cut-off frequency of the waveguide, respectively. Next, a Nanowired SIW isolator is demonstrated. It shows a nonreciprocal isolation of 12 dB (corresponding to 4.4 dB/cm), observed in absence of a DC magnetic field, and achieved through an adequate positioning of ferromagnetic nanowires inside the waveguide cavity.

## 1. Introduction

Porous templates loaded with nanowires have been extensively studied and exploited to fabricate planar and monolithic devices, thanks to the interaction between nanowires and microwave signals. Microwave devices based on nanowires have several advantages over classical components: wide range of used frequencies, stability over temperature, miniaturization through integration into a single substrate. Ferromagnetic nanowire arrays have higher saturation magnetization and ferromagnetic resonance (FMR) than classical ferrites. They can also operate at higher frequencies due to their large aspect ratio.

Owing to those properties, absorbers [1,2], inductors [3], noise suppressors [4] or filters based on electromagnetic band-gap effect [5], have been developed, as well as non-reciprocal devices, including circulators [6], isolators [7] and phase shifters [8]. More recently, the high permittivity of metallic nanowire arrays [9] was used to design slow-wave transmission lines [10], while the double ferromagnetic resonance effect in magnetic nanowires was exploited to design a filter [11]. Different nanowire materials have been combined with coplanar or microstrip topologies.

As outlined in Reference [12], high-quality transmission lines are necessary to match the constraints of low cost and consumption, high density and high operational frequency. Above 30 GHz, interferences and radiation losses deter the performances of classical microstrip and coplanar geometries. Hence, the substrate integrated waveguide (SIW) concept [13] is an interesting solution. Two rows of vertical metallic wires or metallized vias are drilled throughout the entire height of a substrate to obtain a rectangular section of shielded line, allowing to reduce radiation. The presence of wires also prevents spurious interferences by crosstalk due to electromagnetic coupling between adjacent devices in a same circuit. Crosstalk as low as −20 dB [14], −39 dB [15], or even −50 dB [16] are reported for SIW technology. Various active and passive devices can be integrated in the same substrate, including antennas, filters, couplers, power suppliers, for operation up to 180 GHz, and using different kinds of substrates (Printed Circuit Boards (PCB), paper, polymers or alumina) [17,18]. A review and recent developments on SIW antennas can be found in References [19,20,21]. For classical antennas, crosstalk is also a critical isssue since it is responsible for spurious coupling between adjacent patches in an array, resulting into a degradation of radiation performances. Solutions based on metamaterials [22,23] and periodic electromagnetic band gap (EBG) structures [24,25] have been proposed. These solutions will be briefly addressed for SIW antennas in Section 2.3.2.

The combination of nanowires and substrate integrated waveguide (SIW) devices is a promising approach, taking into account their respective advantages [12,26,27]. This concept uses metallic nanowire arrays deposited in a porous alumina template to form the waveguide walls. Then, Cu layers deposited on top and bottom surfaces of the alumina substrate form a simple rectangular waveguide. Various nanowire arrays can be added inside the waveguide cavity, combining different heights, shapes and materials (magnetic or not), to obtain competitive devices. Their realization requires to grow nanowires inside the template in specific positions with a good precision and accuracy.

In this paper we compare two laser-etching processes developed for the precise location of areas hosting the growth of nanowires in a porous alumina membrane. In the first method, the laser opens windows in a top metallic mask, in order to allow the growth in these areas. In the second method, the laser etches the surface of the pores in dedicated areas in order to destroy the porosity and prevent the growth of nanowires during the ECD (electrochemical deposition) process. The two methods are described in the following sections. Next, their application is illustrated with the fabrication of two Nanowired Substrate Integrated Waveguide devices (NSIWs).

## 2. Results and Discussion

### 2.1. Nanowired Template

**Figure 1:**
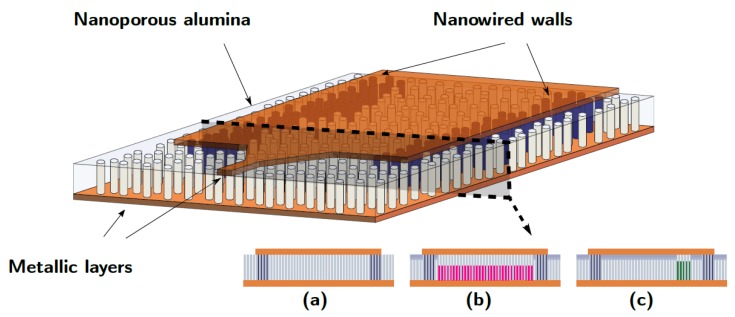
Schematic view of the Nanowire Substrate Integrated Waveguide (NSIW) in the alumina template, and different possible profile sections: (**a**) empty waveguide (**b**) homogeneous filling (**c**) asymmetric filling used for the isolator device. The proper placement of the nanowire arrays is achieved with the laser-assisted process described in Section 2.3.1 or Section 2.3.2.

A schematic of the porous template supporting the Nanowired Substrate Integrated Waveguide is shown in Figure 1. Some regions contain electrodeposited nanowire arrays loaded inside the alumina template. The procedure of electrodepositon is briefly described in Section 3.2. It allows a fine control of the height of deposited nanowires (NWs), while their diameter is fixed by the pore diameter of the membrane, as shown in Section 3.1.

### 2.2. Design of NSIW Devices

The NSIW is constituted of two parallel rows of nanowire arrays separated by a distance *a*, embedded in a nanoporous template covered on both faces by 3 μm thick copper layer (see Figure 1). This structure constitutes a shielded rectangular cavity through which a microwave signal can propagate according to precise modes defined by the classical waveguide theory [28]. This signal only propagates above a cut-off frequency $f_{c}$ depending on the waveguide width *a* and height *b*, on the medium relative permittivity $\varepsilon_{r}$ and permeability $\mu_{r}$ and on the type of mode. For the first modes, $f_{c}$ has the expression:

(1) $$f_{c_{m0}}=c_{0}{\left(2ma\sqrt{\varepsilon_{r}\begin{array}{c} \mu_{r} \end{array}}\right)}^{-1}$$

where $c_{0}$ is the speed of light in vacuum and *m* is the index of the mode. This cut-off frequency constitutes the lower bound of the single mode interval, where no other can create interferences. A frequency range exists where only one mode, referred as TE${}_{10}$ can appear. The latter has the lowest cut-off frequency.

The relative permittivity of nanoporous AAO is given by

(2) $$\varepsilon_{r,AAO}=p\varepsilon_{r,air}+\left(1-p\right)\varepsilon_{r,Al_{2}O_{3}}$$

with *p* the porosity of the alumina template, $\varepsilon_{r,air}=1$ the permittivity of the empty pores, $\varepsilon_{r,Al_{2}O_{3}}=\varepsilon_{a}\left(1-j\tan\delta_{a}\right)$ the permittivity of bulk alumina, $\varepsilon_{a}=9.8$ the dielectric constant of bulk alumina and $\tan\delta_{a}=1.5\times 10^{-2}$ its loss tangent factor.

For the dominant TE${}_{10}$ mode, the magnetic field inside the waveguide has the following expression:

(3a) $$H_{x}\left(x,y,z\right)=A\;\frac{jka}{\pi }sin\frac{\pi x}{a}$$

(3b) $$H_{y}\left(x,y,z\right)=0$$

(3c) $$H_{z}\left(x,y,z\right)=A\;cos\frac{\pi x}{a}$$

where *A* is a constant and $k=2\pi f\sqrt{\varepsilon_{r}\mu_{r}}/c_{0}$ is the propagation constant with *f* the operating frequency. The magnetic field is circurlarly polarized if

(4) $$H_{x}\left(x,y,z\right)=\pm jH_{z}\left(x,y,z\right)$$

This occurs at a precise transversal position $x_{o}$, given by

(5) $$x_{0}=\pm \frac{a}{\pi }\arctan j\frac{\pi }{ka}$$

To obtain an isolator, a ferromagnetic material has to be inserted in the waveguide cavity at position $x_{0}$. Indeed, a circularly polarized magnetic field oscillating in a magnetized medium can induce ferromagnetic resonance: the tensorial permeability $\bar{\bar{\mu }}$ collapse in an effective scalar permeability noted $\mu_{eff}$. This permeability seen by the circularly polarized wave is depending on the direction of propagation of the wave (along positive or negative longitudinal *z*-axis of SIW) and is written

(6) $$\mu_{eff}^{\pm }=\mu \pm \kappa$$

where κ and μ hold for the off-diagonal and diagonal terms of the tensor $\bar{\bar{\mu }}$ describing the permeability of the ferromagnetic nanowire array placed in $x_{0}$. These components are given by [6]

(7) $$\begin{array}{ccc} \mu & = & 1+ph\frac{\left(\omega_{r}+j/\tau \right)\omega_{M}}{{\left(\omega_{r}+j/\tau \right)}^{2}-\omega^{2}}\;\frac{\left(1+m^{2}\right)}{2}\\ \kappa & = & ph\frac{\omega \omega_{M}}{{\left(\omega_{r}+j/\tau \right)}^{2}-\omega^{2}}\;m \end{array}$$

where *h* stands for the nanowire array relative height, *p* for the nanowires packing factor which coincide with the porosity of the alumina template, $\omega_{r}=\omega_{M}\left(1-3p\right)/2$ for the resonance pulsation. $\omega_{M}=\gamma \mu_{0}M_{s}$ is the Larmor pulsation, $M_{s}$ is the saturation magnetization and $m=M/M_{s}$ the normalized remanence. Finally, the material properties γ and τ are the gyromagnetic ratio and the relaxation time, respectively [5,6], while ω=2πf.

Close to the resonance frequency, the circularly polarized wave, propagating towards the negative *z*-axis through the ferromagnetic slab placed inside the waveguide according to Equation (5), will experience a strong attenuation induced by the resonant imaginary part of $\mu_{eff}^{-}$. On the other hand, the wave propagating towards the positive *z*-axis will be preserved since the imaginary part of $\mu_{eff}^{+}$ remains close to zero, inducing almost no attenuation. This is the desired non-reciprocal effect: the wave can easily travel the NSIW loaded with ferromagnetic nanowires in one direction, while being blocked in the other. The ratio between attenuations in the two directions is defined as isolation and is proportional to $\begin{array}{c} ℑ(\mu_{eff}^{-}) \end{array}$.

### 2.3. Laser Etching Processes

#### 2.3.1. Laser Etching of Sacrificial Layer

**Figure 2:**
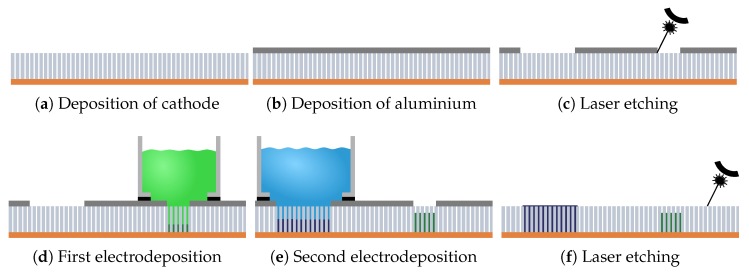
Schematic description of laser patterning process based on the etching of a sacrificial layer (LES) applied to a device containing two materials of different heights. (**a**) Deposition of the copper cathode at the bottom of the alumina membrane. (**b**) Deposition of the 1000 nm thick aluminium layer on the top face of the alumina membrane. (**c**) the laser both defines and etches the desired pattern. (**d**) electrodeposition is used for filling alumina template inside porous regions. The growth area is defined by the etched aluminium while the black rubber is used as a seal. Here, the electrochemical deposition (ECD) is carried out until the nanowires (NWs) reach half of the membrane height. (**e**) Electrodeposition is performed in another area until complete filling of pores and metal overflow. In this scheme, the nanowires are connecting the two metal electrodes. (**f**) Complete removal of the aluminium layer on top face of unfilled porous regions.

This laser patterning process begins with the deposition of the cathode onto the bottom face of the template (Figure 2a). A 1.5 μm thick bottom metallic layer (Cr (5 nm)/Cu (1400 nm)/Au (100 nm)) is deposited by e-beam evaporation. It serves as cathode for the electrodeposition of nanowire arrays and as ground plane of the RF device. A 1 μm thick Al sacrificial layer is then obtained by sputtering onto the top of the AAO membrane in a separate step (Figure 2b). Then the first laser etching step (Figure 2c) is carried out on aluminium. The laser operation is described in Section 3.3. The following laser etching parameters are used: spacing between adjacent lines (2 μm), etching rate (200 mm·m${}^{-1}$) and pulse repetition frequency (40 kHz). After the laser etching, nanowires are grown in the released pores by ECD described in Section 3.2 (Figure 2d,e). Various materials can be deposited in different released areas and different heights can be reached. The ECD of the nanowires is not affected by the aluminium layer on top of the membrane unless they overflow the nanopores. In such a case, a short circuit appears between the cathode and the aluminium layer, rendering electrodeposition in the pores much less effective. Hence, in the case of the pattern used in the process described by the Figure 2, the steps c and b could not have been inverted. If the overfilled nanowires are grown first, it will not be possible to make the second nanowire arrays. Afterwards the aluminium layer is totally removed (Figure 2f). It is worth mentioning that the laser beam cannot pass on the filled sections as it affects the nanowires contained in the template and slightly modify their magnetic properties. The result is an AAO membrane loaded with two nanowires arrays constituted of different materials and having different thicknesses. Finally, a second deposition of metallic layer is performed on the top side of the filled membrane, in order to realise the RF devices via a laser-etching of adequate metallic patterns for RF devices, as will be illustrated in Figures 4a and 5b. For convenience, the method is noted LES for laser etching of sacrificial layer.

The LES process suffers from a lack of stability: uncontrolled small variations of the laser power generate dramatic changes in the efficiency of the aluminium etching process. Reductions induce a risk of an underetching of the sacrificial layer preventing the ECD. On the other hand, the template surface porosity can be destroyed by a too large laser power, preventing again the ECD. Section 2.3.2 proposes a simple and flexible laser-assisted procedure, based on the localized controlled destruction of the surface porosity. The electrolyte only enters in non-destroyed porous areas and small variations of laser power have no effect on their definition.

#### 2.3.2. Laser Etching of Pores

In order to overcome the issues presented above with the LES process, we propose here a second procedure enabling the controlled destruction of the porous template surface over well-defined regions. In this scheme, the electrolyte only enters in non-damaged porous areas thus providing a simple and flexible method for a spatial control of NW growth. This process is noted LEP for laser etching of pores. Moreover, the ECD process is not affected by the small variations of laser power. Also, no additional etching step has to be performed, thus keeping the NWs far from laser beam. Finally, it also allows complex patterns to be generated for various microwave devices.

**Figure 3:**
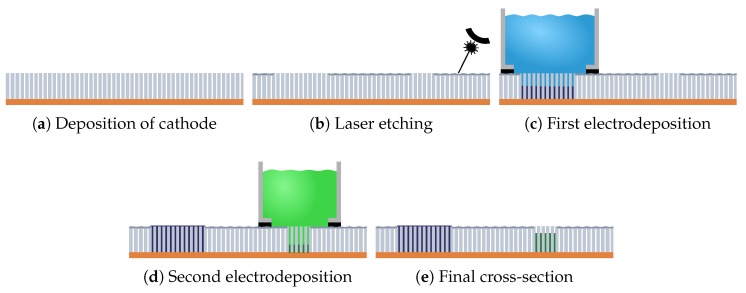
Laser process based on the Etching of the template surface Porosity (LEP), for a device including two materials of different heights. (**a**) E-beam deposition of Copper cathode. (**b**) Localised laser destruction of porosity on the top face. (**c**) ECD of first material until overflowing. (**d**) ECD of second material until a partial height in unclogged pores. Unlike the LES method, the order of the ECD can be inverted. (**e**) Final cross-section of the AAO membrane.

As in the previous procedure, the first experimental step is the deposition of the bottom metallic layer, which will act as a cathode for nanowires growth and as a ground plane for RF measurements (Figure 3a). Then, we carry out a laser etching of porous areas on the top face of the membrane (Figure 3b). In order to damage sufficiently the AAO template surface without removing a substantial thickness, we make three passes at a power of 0.10 W, a pulse frequency of 40 kHz, with a speed of 200 mm/s, and a distance between the lines of 5 μm. These parameters allow a homogeneous distribution of the laser spots in the two directions of the etched layer. After the etching, a small thickness of alumina has been removed in the illuminated parts. These parameters are a tradeoff between several constraints. First, the etching must not sublimate the alumina over a too large thickness. This could be a problem for the RF device design. Secondly, the power must remain relatively small enough to not degrade the cathode through the alumina. A damaged cathode could also cause RF losses. These first two conditions require the use of a relatively low laser power. Hence, passing several times is necessary to ensure the clogging of the pores. Finally, the areas to be engraved are larger than in the case of the first method. It is therefore necessary to have a high engraving speed and a relatively low path density so that engraving does not take too long.

After laser etching, the nanowires growth by ECD takes only place in the pores that have not been damaged by laser beam. It allows then a precise control of their positioning. Different materials can be deposited inside the same template using several ECD steps. The height of the nanowires can also be controlled by the deposition time to meet the needs. In contrast to the former LES method, the ECD in the LEP process may be continued after overfilling of the pores. As a result, the order in which the ECD steps are conducted to create the example pattern reported in Figure 3 can be inverted. Finally, as in Section 2.3.1, RF patterns are laser-etched in the top metallic layer.

**Figure 4:**
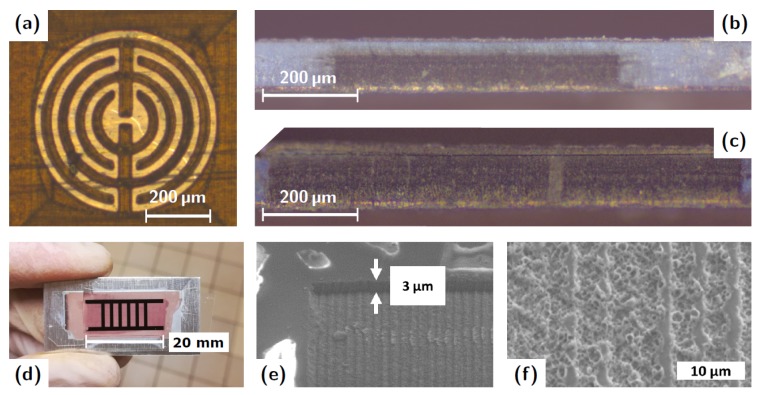
Devices realized with LEP process, at different steps. (**a**) Optical microscope (OM) image of IDEs after etching and before ECD. (**b**) OM image of an AAO template filled with NiFe nanowires at 70% of thickness of the template. (**c**) OM image of a template filled with Cu nanowires at 100%. (**d**) CCD camera image of a SIW device with EBG effect after ECD. Black areas are the nanowire-filled sections. The width between the two long lines forming the walls is 6 mm. (**e**) Scanning electron microscope (SEM) image at the edge of the etched area. The sublimated thickness is about 3 μm. (**f**) SEM image of the rough surface of the etched area.

In Figure 4, several images of results obtained with the LEP process are presented. In Figure 4a, an example of interdigitated electrodes (IDE) pattern after etching but before ECD is given. The etched part is darker while the bright color indicates the un-attacked pores ready for ECD. This shows an example of complex pattern of grown nanowires that can be created. Figure 4b,c are two optical microscope (OM) images of cross section of a 70% filled and a 100% filled membranes. We see the sharp transition between filled (black) and unfilled (blue) regions and the uniform filling along the whole cross-section. A CCD photograph of a microwave device (substrate integrated waveguide (SIW) with a EBG filter effect) is given Figure 4d. The darker areas are filled with nanowires. The width between the walls of the waveguide (i.e., the two long nanowired rows) is 6 mm. Six rectangular nanowired areas are posted in the guide in order to create an Electromagnetic Band Gap effect similar to Reference [5]. Finally, the two SEM images in Figure 4e,f give a view of the surface after the etching: the surface porosity is completely destroyed.

As briefly mentioned in the introduction, EBG structures are relevant in the filters and antennas fields. The same statement applies for the SIW technology. In References [29,30], a 2-D periodic EBG structure was implemented around an array of 4 SIW antennas to reduce spurious coupling and enhance the performances. Topologies of EBG SIW filters [31,32] or delay lines [33] were also proposed. SIW EBG filters and antennas could be easily implemented using nanowires and the LES or LEP method presented in this paper.

#### 2.3.3. Comparison between LES and LEP Processes

The LES process presented in Section 2.3.1 suffers from several issues. First, its feasibility is sensitive to the stability of the laser source power. Indeed the growing areas are determined by the etching of the sacrificial layer. If the power is too low, the metal layer will be underetched, and remains a barrier against the ECD. Conversely, if the power is too high, the porosity of the membrane is destroyed, preventing again the ECD. In the two cases a fine tuning and readjustement of the laser power is necesssay, affecting the good reproducibility of the process. On the contrary in the LEP method presented in Section 2.3.2 the laser power does not affect the ECD, since it aims to destroy only the pore areas where ECD must be prevented.

The second drawback of the LES method of Section 2.3.1 is related to the thickness and nature of the sacrificial layer necessary to prevent ECD in the areas where NWs are not needed. We used an aluminum thickness of 1000 nm to completely seal the pores of the templates in areas where ECD must be avoided. Etching such a thickness requires to use a high number of passes of the beam over a same track since the laser power cannot be increased too much as explained above. Hence, the sources of errors are multiplied and the irreproducibility is enhanced.This drawback is not present in the second method LEP since there is no need for etching of a sacrificial layer before ECD.

Another advantage of the LEP method in Section 2.3.2 is that the order of the various ECDs is not important. The ECD of overfilled nanowires in some areas of the template—which is mandatory for the realisation of NSIW devices illustrated in Figure 1—does not prevent next ECDs of nanowires having smaller height.

Finally, as the initial sacrifial layer is not present in the LEP method, there is no risk to damage the magnetic properties since the final etching step of Figure 2f is absent. Our studies have indeed shown that the laser beam illuminating a nanowired area dramatically affects the apparent saturation magnetisation of the nanowires, lowering its value by at least 20%. This is due to a significant loss of magnetic material during laser ablation, which may be critical for the operation of microwave nonreciprocal devices.

For the sake of completeness Table 1 provides an additional comparison between laser process and classical lithography technique.

### 2.4. Microwave Devices

#### 2.4.1. NSIW Line

The geometry of the NSIW line is depicted in Figure 1. The nanoporous alumina template described in Section 3.1 is filled with ferromagnetic nanowires (NWs) according to the ECD process in Section 3.2. Figure 1b shows the cross-section of the NSIW homogeneously filled with NWs, while Figure 1c corresponds to an NSIW filled in selective areas using the LEP process described in Section 2.3.2.

**Figure 5:**
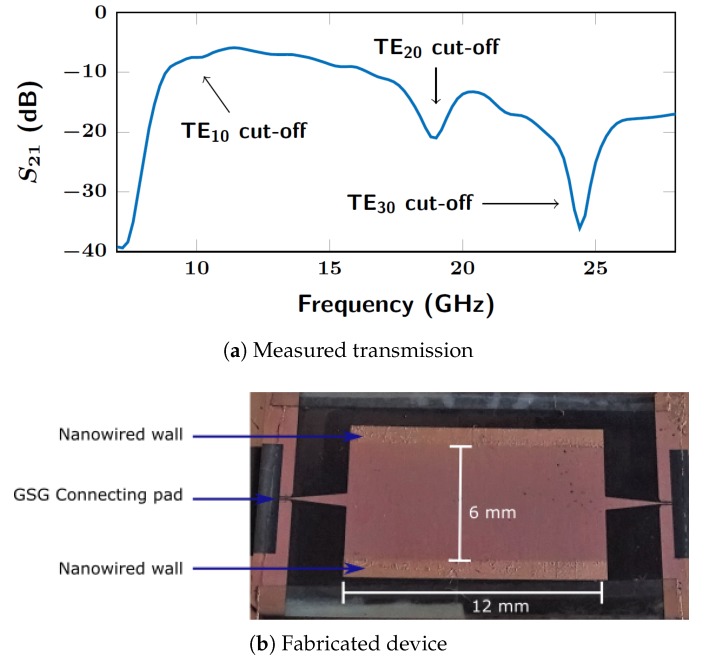
(**a**) Measured transmission through a 12 mm long NSIW. Two walls made of Cu nanowires define a 6 mm wide cavity guiding the signal and inducing a $TE_{10}$ cut-off effect at 8.5 GHz, as predicted by the theory (1). (**b**) photograph of the fabricated device, showing the NSIW cavity of length 12 mm embedded between two triangular tapered microstrip transitions, each connected to a rectangular contact pad for the coplanar waveguide (CPW) measuring probe.

The measured transmission S${}_{21}$ through the empty NSIW (Figure 1a) is shown in Figure 5. The measurement method is described in Section 3.4. Shielding walls are made with Cu nanowires, the waveguide cavity size is 6 × 12 mm${}^{2}$. The cut-offs of the 3 first modes TE${}_{10}$, TE${}_{20}$ and TE${}_{30}$ appear at the values predicted by the theory (1): 8.5 GHz, 17 GHz and 25 GHz.

#### 2.4.2. NSIW Isolator

**Figure 6:**
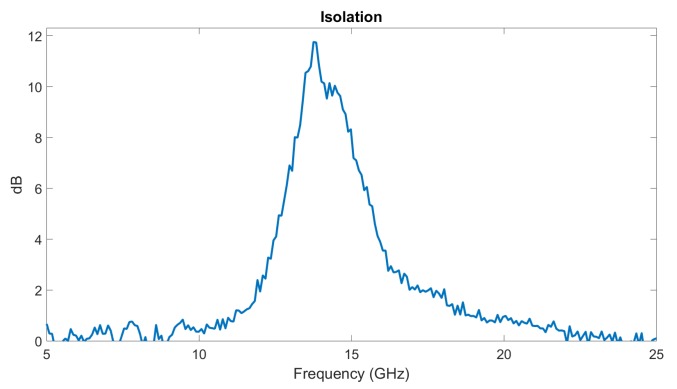
Difference between the forward S${}_{21}$ and backward S${}_{12}$ transmissions, i.e., isolation, obtained through a NSIW isolator. The non-reciprocal effect is created by an area of NiFe nanowires asymmetrically placed inside the waveguide cavity (see Figure 1c). The NSIW isolator is fabricated using the LEP process with 1.5 mm-wide NiFe area.

An NSIW isolator was also designed and fabricated using the LEP process in Section 2.3.2, and measured. The cavity is 6 mm-wide and 20 mm-long. A 10 mm-long and 1.5 mm-large area of ferromagnetic NiFe nanowire arrays was grown inside the cavity (see Figure 1c) at the transversal position given by (5), $x_{0}$ = 1.6 mm. The relative height of nanowires is 70%. We used saturation magnetization $M_{s}$ = 1000 kA·m${}^{-1}$, remanence factor m = 0.8, the gyromagnetic factor γ=3.08×$10^{10}$ s·A·kg${}^{-1}$ and relaxation time τ = 7 × 10${}^{11}$ s. The ferromagnetic nanowired-filled area inside the waveguide is 10 × 1.5 mm${}^{2}$ = 15 mm${}^{2}$. The difference between the forward S${}_{21}$ and backward S${}_{12}$ transmissions, i.e., the isolation, is shown on Figure 6. A maximum occurs at 13 GHz, which is induced by the ferromagnetic resonance of NiFe material, and is the signature of a nonreciprocal propagation through the device, since S${}_{21}$ and S${}_{12}$ are significantly different (12 dB).

The LEP process preserves the porosity in areas where ECD occurs, hence the amount of nanowires in the template is uniform over the whole area of the membrane, other said the targed filling factor of 70% is reached uniformly in the template. As a consequence, the value of permeability $\begin{array}{c} ℑ(\mu_{eff}^{-}) \end{array}$ (6) remains well controlled, as well as finally the level of isolation.

The insertion losses observed in Figure 5 (8 dB at 12 GHz, corresponding to 4.4 dB/cm) are comparable or lower than other technologies/topologies, as shown Table 2. They should be significantly decreased by further improvement of the devices. Indeed, the upper and lower Cu layers deposited on the two faces of the AAO membrane can be thickened to increase the shielding effect. Also, the amount of magnetic material placed inside the isolator can be increased by enlarging the width of the NiFe NW strip. All those improvements will enhance significantly the performances of NSIW devices in terms of insertion losses in order to achieve insertion losses lower than 1 dB/cm.

## 3. Materials and Methods

### 3.1. Nanoporous Template

**Figure 7:**
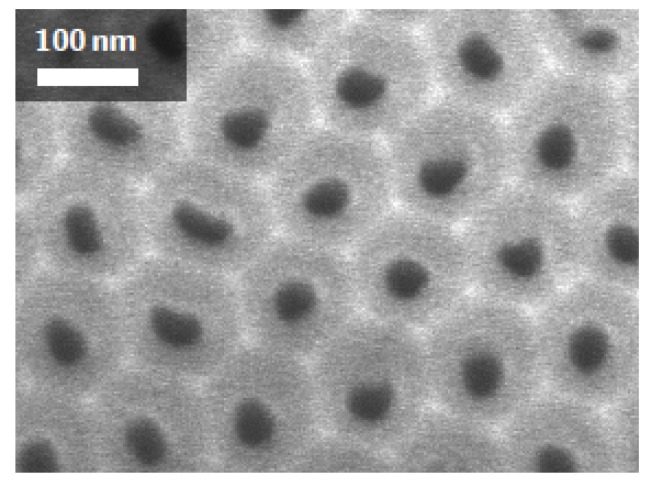
SEM images of a self-supported AAO templates with pore diameter of 40 nm, interpore distance of 125 nm, and thickness of 100 μm.

The porous templates used for this work are anodized aluminium oxide membranes (AAO) from Smart Membranes GmbH (Figure 7). The pore diameter is 40 nm, the distance between pores is 125 nm and the thickness is 100 μm.

### 3.2. Electrochemical Deposition Process (ECD)

**Figure 8:**
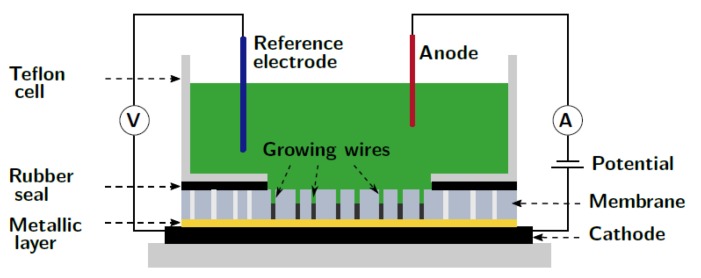
Schematic illustration of the electrodeposition cell using the three-electrodes configuration (i.e., three-layered cathode, Pt anode and AgCl reference). The membrane is covered by the Teflon cell containing the electrolyte, over an area where the pores are opened. A piece of rubber is inserted for ensuring the sealing. Adapted from Reference [39].

Prior to the ECD process, a thick metallic layer is deposited on one face of the AAO template, to completely cover the nanopores and play the role of cathode during the electrodeposition. The usual cathode is a three-layer (Cr (5 nm)/Cu (1400 nm)/Au (100 nm)) deposited by e-beam evaporator. A schematic representation of the experimental set-up is shown in Figure 8. The alumina membrane is placed in a Teflon cell that contains the electrolytic solution. We used an AgCl reference electrode and a Pt wire as anode The three-electrodes are connected to a EG & G Princeton Applied Research potentiostat.

Different materials were deposited inside the same porous template using successive electroplating steps. The height of the nanowires was controlled by the deposition time (see Figure 4), modifying subsequently the permittivity ε of the filled template sections [9]. In this work, we used two different electrolytic solutions:

- for NiFe nanowires:
  (1 M NiSO${}_{4}$ + 0.02 M FeSO${}_{4}$ + 0.5 M H${}_{3}$BO${}_{3}$), V = −1.05 V
- for Cu nanowires:
  (1 M Cu basis + 0.08 M H${}_{2}$SO${}_{4}$ + chloride basis + organic additives), V = −0.015 V
  The detailed composition of the Cu solution is a property of Sigma-Aldrich, Inc.

### 3.3. Pico-Second Laser System

The used laser is a Q-Switch picosecond laser assembled by Oxford Lasers Ltd. [40], powered with laser source fabricated by Coherent, Inc, operating in the ultraviolet (UV) range at 355 nm wavelength. Its use was previously illustrated for the micromachining of carbonated nanostructures [41]. This “pico-second” corresponds to the order of magnitude of the duration of the laser pulses and is therefore not directly related to their frequency. Due to the UV light absorption difference between the aluminium and alumina, alumina is less damaged with the etching. The pulses repetition frequency is basically fixed at 200 kHz and the 100% laser power is close to 4 W. These parameters can be lowered to meet the needs. To fully remove aluminium, a power of 0.05 W and 3 passes are necessary and these parameters prevent cathode damage. The laser etches clean and sharp edges of metal. The resulting aluminium walls show micron size surface roughness and the pores are totally opened in the etched areas [27], so that the electrolyte can easily penetrate into the AAO template for the subsequent EDC.

**Figure 9:**
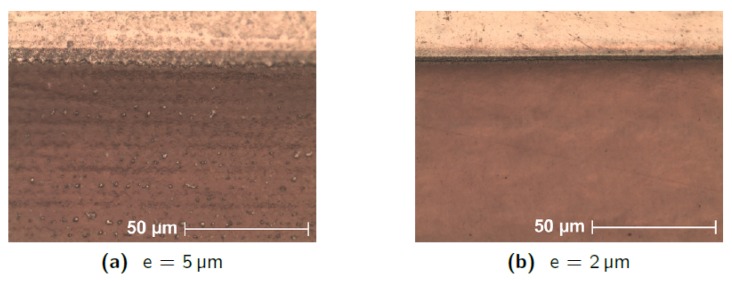
Optical images after etching of an Al 1000 nm layer taken with *e* = 5 μm (**a**) and *e* = 2 μm (**b**). The white material is aluminium and the brown color comes from the cathode seen through the AAO. A greater distance *e* allows horizontal lines to appear, parallel to the engraving paths, separated from 5 μm, indicating insufficient overlap. If *e* is reduced, recovery is better and homogeneity is significantly improved. The definition of the edge of the cleaned area is also much better with *e* = 2 μm. The etching parameters are $f_{r}$ = 100 kHz, *P* = 0.05 W, *n* = 3.

**Figure 10:**
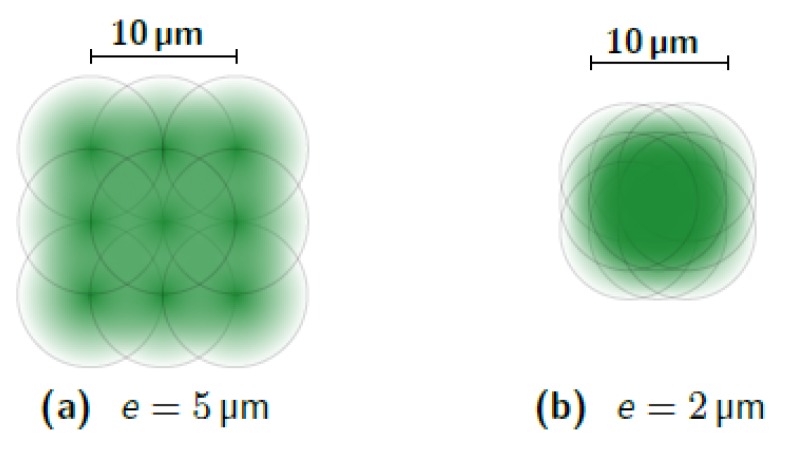
Schematic distribution of laser power on two illuminated areas with two values of *e*, the distance between spots. The intensity in the spot respects a Gaussian distribution. By decreasing *e*, the overlap and the homogeneity of the etching are increased but the etched surface is reduced.

The selection of the laser parameters used to remove a 1000 nm thick layer of aluminium deposited on a nanonanoporous alumina membrane without damaging it is as follows. First, for simplicity, vertical defocusing is set to zero because its effects are difficult to predict. We aim to have the smoothest and homogeneous possible etching. This is possible by imposing a uniform distribution of laser spots in the horizontal plane, in both X and Y directions. The distance between the lines—denoted *e*—must therefore correspond to the distance between two spots on the same line. This implies the relation:

(8) $$e=\frac{v}{f_{r}}$$

where *v* is the speed of the spot and $f_{r}$ is the pulse repetition frequency. The distance *e* is set first. Considering that the diameter of the laser spot is 10 μm and that an overlap is necessary to ensure a correct ablation of the aluminium, we start by imposing *e* = 5 μm. Several tests performed with this value have shown that, for a thickness of 1000 nm, the induced overlap is not sufficient (see Figure 9a). Indeed, as the passes go by, the lines on which the spot center pass are more intensely engraved than the areas between these lines. This result is due to two effects: the inaccuracy of the laser spot longitudinal position on the one hand, and the non-homogeneity of the laser power on the spot surface on the other hand. The power in the laser beam follows a Gaussian distribution. In order to increase the recovery and reduce the impact of these two factors, the value of 2 μm was chosen for *e*. This provides better homogeneity in the etching (see Figure 9b and Figure 10). Experience shows that if the laser spot moves too fast, the definition of the followed path is not accurate, especially around corners and half-turns. This definition comes acceptable when the speed approaches 300 mm/s. We chose 200 mm/s, which is a commonly used value. Hence, according to Equation (8), the frequency $f_{r}$ must be 100 kHz.

The power and number of passes still have to be determined. Different experiments have led to the conclusion that passing three times (*n* = 3) over the aluminium layer, with a power corresponding to 4% of the internal power (which corresponds to 0.05 W if $f_{r}$ is 100 kHz) provides very good results. Using lower power does not effectively sublimate the aluminium while reducing the number of passes doesn’t give the possibility to finely control the engraved depth. Figure 9 was obtained on a square engraved with these parameters.

### 3.4. Microwave Characterization

The standard microwave measurements were conducted with a microwave prober PM8 built by FormFactor, Inc. The microwave transmission is measured with a vector network analyser (VNA) model N5247A PNA-X from Agilent Technologies. The devices under test (DUTs) were connected thanks to coplanar GSG probes that had a 150 μm pitch, fixed on the prober and connected to the VNA via high performances semi-rigid RF cables. GSG probes are used to excite a tapered microstrip line feeding the NSIW devices. The coplanar waveguide (CPW) ports and the microstrip lines are designed to be matched to 50 Ω, while the dimensions of the tapered transitions from microstrip to SIW have been optimized numerically to prevent the generation of spurious propagation modes. The VNA measures the S-parameters of the devices at 401 frequency points homogeneously ranged from 100 MHz up to 40 GHz. Prior to measurements, the setup was calibrated with a SOLT method [42] using a standard planar calibration kit. The de-embedding is executed by the VNA itself. This way, the reference planes between which the transmission is determined are moved at the end of the two RF probes.

## 4. Conclusions

Various structures based on nanoporous templates have been studied in the past. In particular several devices were developed using templates filled with ferromagnetic nanowires for operation in the microwave frequency range. This paper compares two laser-assisted processes developed by the authors for the fabrication of various microwave devices exploiting nanoporous alumina templates filled with ferromagnetic nanowires. The pros and cons of each process are discussed. The efficiency of the laser-assisted process is demonstrated through the realization of various devices, showing performances in line with the literature. They combine ferromagnetic nanowires with the Substrate Integrated Waveguide topology. The present work paves the road towards the realization of tunable SIW antennas and filters, by exploing the tunability of the FMR phenomenon via an external DC magnetic field, as we already demonstrated for the microstrip technology [11].

## Figures and Tables

**Table 1:** Comparison between lithography and laser processes.

Technology	Lithography	Laser
accuracy	good (<100 nm)	good (<1000 nm)
reproducibility	good (mastered process)	poor for LES (unstability of power source)
cost	high (fabrication of masks)	cheaper
handling	intricate	easy and fast
versatility	poor (1 mask needed per set of devices)	easy reconfigurable for each size and topology
risks	clogging of pores by resins	impact of rugosity on RF losses

**Table 2:** Comparison of insertion losses (IL).

Ref.	Topology	Technology	f (GHz)	IL (dB/cm)
[34]	CPW	Ni${}_{x}$Si${}_{y}$/Si	10	2
[35]	CPW	MMIC	20	5
[36]	SIW	LTCC	50	2.53
[37]	SIW	LTCC	38	4
[10]	microstrip	NW in AAO	60	7
[38]	SIW	NW in AAO	20	7.4
present work	SIW	NW in AAO	12	4.4

## Abbreviations

The following abbreviations are used in this manuscript:

AAO	Anodic Aluminium Oxide
CCD	Charge-Coupled-Device
CPW	Coplanar Waveguide
Cu	Copper
DUT	Device Under Test
EBG	Electromagnetic Band Gap
ECD	Electro-Chemical Deposition
GSG	Ground-Signal-Ground
IDE	Interdigitated Electrode
NiFe	nickel-iron alloy
MMIC	Monolithic Microwave Integrated Circuit
LEP	Laser Etching of Pores
LES	Laser Etching of Sacrificial layer
LTTC	Low Temperature Co-fired Ceramics
NW	Nanowire
NSIW	Nanowired Substrate Integrated Waveguide
OM	Optical Microscope
PCB	Printed Circuits Boards
RF	Radio Frequency
SEM	Scanning Electron Microscope
SOLT	Short-Open-Line-Thru
SIW	Substrate Integrated Waveguide
VNA	Vector Network Analyzer
